# Supplementary material for: Assessment of STAT4 Variants and Risk of Hepatocellular Carcinoma in Latin Americans and Europeans
Source: Cancers (Basel). 2023 Sep 12;15(18):4530. doi: 10.3390/cancers15184530 (PMC10527221; doi:10.3390/cancers15184530)
Supplement: Supplementary file 1 [file cancers-15-04530-s001.zip › cancers-2575825-supplementary.pdf]

Supplementary Table S1. Table with previous studies on STAT4 rs7574865 and liver pathology

| AUTHORS | YEAR | RISK ALLELE | NUMBER OF PATIENTS | PAPER COUNTRY | INVESTIGATED DISEASE |
|---|---|---|---|---|---|
| Wang et al. [1] | 2022 | G | 1000 | CHINA | HCC |
| Zhong et al. [2] | 2021 | G | 1262 | CHINA | HCC - 74% chronic HBV |
| Chao et al. [3] | 2021 | no association | 1011 | CHINA | HBV HCC |
| Chen et al. [4] | 2015 | G | 3521 | CHINA | HCC - 82% chronic HBV |
| Qi et al. [5] | 2022 | G | 302 | CHINA | HBV |
| Limothai et al. [6] | 2022 | G | 261 | THAILAND | HBV |
| Li et al. [7] | 2021 | G | 1823 | CHINA | HBV |
| Jalil et al. [8] | 2020 | G | 297 | PAKISTAN | HBV |
| Chen et al. [7] | 2020 | G | 1623 | CHINA | HBV |
| Chen et al. [9] | 2019 | G | 1823 | CHINA | HBV |
| El Sharkawi et al. [10] | 2018 | G | 1095 | AUSTRALIA/EGYPT | HBV fibrosis |
| Jiang et al. [11] | 2016 | G | 466 | CHINA | HBV |
| Lu et al. [41] | 2015 | G | 576 | CHINA | HBV |
| Jiang et al. [49] | 2015 | G | 3313 | CHINA | HBV |
| Liao et al. [50] | 2015 | G only in Tibetans | 1341 | CHINA | HBV |
| Yang et al. [20] | 2022 | G | 3151 | CHINA | HBV HCC |
| Ali N et al. [51] | 2021 | GT | 200 | EGYPT | HCV HCC |
| Yang et al. [52] | 2019 | G | 3041 | EUROPE | HBV HCC |
| Chanthra et al. [53] | 2015 | G | 582 | THAILAND | HBV HCC |
| Kim et al. [27] | 2015 | no association | 3838 | KOREA | HBV HCC |
| Jiang et al. [14] | 2013 | G | 11799 | CHINA | HBV HCC |
| Clark et al. [28] | 2013 | no association | 667 | VIETNAM | HBV HCC |
| Chen et al. [22] | 2013 | no association | 1278 | CHINA | HBV HCC |
| Shi et al. [54] | 2019 | G | 13769 | CHINA | HBV HCC |
| Xiao et al. [55] | 2018 | G | 9174 | CHINA | HCC |
| Zhang et al. [25] | 2017 | G | 21334 | CHINA | HCC |
| Jiang et al. [26] | 2016 | G | 3033 | CHINA | HBV |
| Zhao et al. [27] | 2015 | G | 12244 | CHINA | HBV HCC |
| Liao et al.[28] | 2014 | no association | 6160 | CHINA | HBV vs HCC |

Supplementary Table S2. Comparing G allele frequency of cirrhotic patients to patients without cirrhosis and divided for Latin Americans (LA), and Europeans (EU)

| | OR | | CI 95% | | P | |
|---|---|---|---|---|---|---|
| | LA | EU | LA | EU | LA | EU |
| CIRRHOTICS vs NON-CIRRHOTICS | 1.164 | 1.384 | 0.727-1.865 | 0.838-2.285 | 0.554 | 0.249 |

**References**


1. Wang, C.; Gao, N.; Yang, L.; Guo, Y.; Fang, Y.; Wang, T.; Xu, C.; Li, G. fang; Zhou, J.; Zhang, Y.; et al. Stat4 Rs7574865 Polymorphism Promotes the Occurrence and Progression of Hepatocellular Carcinoma via the Stat4/CYP2E1/FGL2 Pathway. *Cell Death Dis* **2022**, *13*, doi:10.1038/S41419-022-04584-4.

2. Zhong, X.; Luo, M.; Wu, Y.; Zhou, X.; Yu, X.; Liu, L.; Chen, S. Genetic Variants in STAT4 and Their Interactions with Environmental Factors for the Incidence of Hepatocellular Carcinoma. *Cancer Biomarkers* **2021**, *32*, 3–9, doi:10.3233/CBM-203162.
3. Chao, X.; Wu, J.; Zhang, W.; Feng, X.; Zhao, L.; Huang, F.; Jiang, C. A New Discovery of STAT4 Single Nucleotide Polymorphisms Associated with Hepatocellular Carcinoma Risk in Chinese Han Population: A Case-Control Study. *Biosci Rep* **2021**, doi:10.1042/BSR20210124.
4. Chen, W.; Wang, M.; Zhang, Z.; Tang, H.; Zuo, X.; Meng, X.; Xiong, M.; Zhou, F.; Liang, B.; Dai, F.; et al. Replication the Association of 2q32.2-Q32.3 and 14q32.11 with Hepatocellular Carcinoma. *Gene* **2015**, *561*, 63–67, doi:10.1016/j.gene.2015.02.006.
5. Qi, X.; Li, F.; Zhang, Y.; Zhu, H.; Yang, F.; Li, X.; Jiang, X.; Chen, L.; Huang, Y.; Zhang, J. STAT4 Genetic Polymorphism Significantly Affected HBeAg Seroconversion in HBeAg-Positive Chronic Hepatitis B Patients Receiving Peginterferon-$\alpha$ Therapy: A Prospective Cohort Study in China. *J Med Virol* **2022**, *94*, 4449–4458, doi:10.1002/JMV.27880.
6. Limothai, U.; Chuaypen, N.; Poovorawan, K.; Poovorawan, Y.; Tangkijvanich, P. Genetic Variation in STAT4 Is Associated with Treatment Response to Pegylated Interferon in Patients with Chronic Hepatitis B. *Asian Pac J Allergy Immunol* **2022**, *40*, 87–93, doi:10.12932/AP-020419-0533.
7. Chen, H.; Sun, J.; Zhou, B.; Peng, J.; Xie, Q.; Liang, X.; Fan, R.; Conran, C.; Xu, J.; Ji, Y.; et al. A Missense Variant in Complement Factor B (CFB) Is a Potential Predictor of 24-Week off-Treatment Response to PegIFN$\alpha$ Therapy in Chinese HBeAg-Positive Chronic Hepatitis B Patients. *Aliment Pharmacol Ther* **2020**, *51*, 469–478, doi:10.1111/APT.15624.
8. Jalil, I.; Arshad, M.; Khan, S.; Dasti, J.I. The STAT4 and Not the IFNL3 Variant Is Associated with Hepatitis B Virus Clearance in a Population from the Khyber Pakhtunkhwa Region of Pakistan. *Arab J Gastroenterol* **2020**, *21*, 91–94, doi:10.1016/J.AJG.2020.04.011.
9. Chen, H.; Sun, J.; Zhou, B.; Xie, Q.; Liang, X.; Fan, R.; Conran, C.; Xu, J.; Ji, Y.; Zhang, X.; et al. Variants in STAT4 Associated With Cure of Chronic HBV Infection in HBeAg-Positive Patients Treated With Pegylated Interferon-Alpha. *Clin Gastroenterol Hepatol* **2020**, *18*, 196-204.e8, doi:10.1016/J.CGH.2019.04.044.
10. El Sharkawy, R.; Thabet, K.; Lampertico, P.; Petta, S.; Mangia, A.; Berg, T.; Metwally, M.; Bayoumi, A.; Boonstra, A.; Brouwer, W.P.; et al. A STAT4 Variant Increases Liver Fibrosis Risk in Caucasian Patients with Chronic Hepatitis B. *Aliment Pharmacol Ther* **2018**, *48*, 564–573, doi:10.1111/APT.14866.
11. Jiang, D.K.; Wu, X.; Qian, J.; Ma, X.P.; Yang, J.; Li, Z.; Wang, R.; Sun, L.; Liu, F.; Zhang, P.; et al. Genetic Variation in STAT4 Predicts Response to Interferon-$\alpha$ Therapy for Hepatitis B e Antigen-Positive Chronic Hepatitis B. *Hepatology* **2016**, *63*, 1102–1111, doi:10.1002/HEP.28423.
12. Lu, Y.; Zhu, Y.; Peng, J.; Wang, X.; Wang, F.; Sun, Z. STAT4 Genetic Polymorphisms Association with Spontaneous Clearance of Hepatitis B Virus Infection. *Immunol Res* **2015**, *62*, 146–152, doi:10.1007/S12026-015-8645-1.
13. Jiang, D.K.; Ma, X.P.; Wu, X.; Peng, L.; Yin, J.; Dan, Y.; Huang, H.X.; Ding, D.L.; Zhang, L.Y.; Shi, Z.; et al. Genetic Variations in STAT4,C2,HLA-DRB1 and HLA-DQ Associated with Risk of Hepatitis B Virus-Related Liver Cirrhosis. *Sci Rep* **2015**, *5*, doi:10.1038/SREP16278.
14. Liao, Y.; Cai, B.; Li, Y.; Chen, J.; Ying, B.; Tao, C.; Zhao, M.; Ba, Z.; Zhang, Z.; Wang, L. Association of HLA-DP/DQ, STAT4 and IL-28B Variants with HBV Viral Clearance in Tibetans and Uygurs in China. *Liver Int* **2015**, *35*, 886–896, doi:10.1111/LIV.12643.
15. Yang, C.; Chen, H.; Zhou, B.; Yin, J.; Cao, G.; Hou, J.; Jiang, | Deke; Jiang, D. The Effects of the Interactions of STAT4 Rs7574865 with HBV Mutations on the Risk of Hepatocellular Carcinoma. **2022**, doi:10.1002/mc.23449.
16. Ali, N.A.; Hamdy, N.M.; Gibriel, A.A.; EL Mesallamy, H.O. Investigation of the Relationship between CTLA4 and the Tumor Suppressor RASSF1A and the Possible Mediating Role of STAT4 in a Cohort of Egyptian Patients Infected with Hepatitis C Virus with and without Hepatocellular Carcinoma. *Arch Virol* **2021**, *166*, 1643–1651, doi:10.1007/S00705-021-04981-8.
17. Yang, J.; Trépo, E.; Nahon, P.; Cao, Q.; Moreno, C.; Letouzé, E.; Imbeaud, S.; Gustot, T.; Deviere, J.; Debette, S.; et al. PNPLA3 and TM6SF2 Variants as Risk Factors of Hepatocellular Carcinoma across Various Etiologies and Severity of Underlying Liver Diseases. *Int J Cancer* **2019**, *144*, 533–544, doi:10.1002/IJC.31910.
18. Chanthra, N.; Payungporn, S.; Chuaypen, N.; Piratanantatavorn, K.; Pinjaroen, N.; Poovorawan, Y.; Tangkijvanich, P. Single Nucleotide Polymorphisms in STAT3 and STAT4 and Risk of Hepatocellular Carcinoma in Thai Patients with Chronic Hepatitis B. *Asian Pacific Journal of Cancer Prevention* **2016**, *16*, 8405–8410, doi:10.7314/APJCP.2015.16.18.8405.

19. Kim, L.H.; Cheong, H.S.; Namgoong, S.; Kim, J.O.; Kim, J.H.; Park, B.L.; Cho, S.W.; Park, N.H.; Cheong, J.Y.; Koh, I.S.; et al. Replication of Genome Wide Association Studies on Hepatocellular Carcinoma Susceptibility Loci of STAT4 and HLA-DQ in a Korean Population. *Infect Genet Evol* **2015**, *33*, 72–76, doi:10.1016/J.MEEGID.2015.04.013.
20. Jiang, D.-K.; Sun, J.; Cao, G.; Liu, Y.; Lin, D.; Gao, Y.-Z.; Ren, W.-H.; Long, X.-D.; Zhang, H.; Ma, X.-P.; et al. Genetic Variants in STAT4 and HLA-DQ Genes Confer Risk of Hepatitis B Virus–Related Hepatocellular Carcinoma. *Nat Genet* **2013**, *45*, 72–75, doi:10.1038/ng.2483.
21. Clark, A.; Gerlach, F.; Tong, H. van; Hoan, N.X.; Song, L.H.; Toan, N.L.; Bock, C.-T.; Kremsner, P.G.; Velavan, T.P. A Trivial Role of STAT4 Variant in Chronic Hepatitis B Induced Hepatocellular Carcinoma. *Infection, Genetics and Evolution* **2013**, *18*, 257–261, doi:10.1016/j.meegid.2013.05.025.
22. Chen, K.; Shi, W.; Xin, Z.; Wang, H.; Zhu, X.; Wu, X.; Li, Z.; Li, H.; Liu, Y. Replication of Genome Wide Association Studies on Hepatocellular Carcinoma Susceptibility Loci in a Chinese Population. *PLoS One* **2013**, *8*, e77315, doi:10.1371/journal.pone.0077315.
23. Shi, H.; He, H.; Ojha, S.C.; Sun, C.; Fu, J.; Yan, M.; Deng, C.; Sheng, Y. Association of STAT3 and STAT4 Polymorphisms with Susceptibility to Chronic Hepatitis B Virus Infection and Risk of Hepatocellular Carcinoma: A Meta-Analysis. *Biosci Rep* **2019**, *39*, 20190783, doi:10.1042/BSR20190783.
24. Xiao, Y.; Liu, G.; Gong, L. Systematic Review and Meta-Analysis on the Association between Polymorphisms in Genes of IL-12 Signaling Pathway and Hepatocellular Carcinoma Risk. *J Cancer* **2018**, *9*, 3583–3592, doi:10.7150/JCA.26419.
25. Zhang, L.; Xu, K.; Liu, C.; Chen, J. Meta-Analysis Reveals an Association between Signal Transducer and Activator of Transcription-4 Polymorphism and Hepatocellular Carcinoma Risk. *Hepatology Research* **2017**, *47*, 303–311, doi:10.1111/hepr.12733.
26. Jiang, X.; Su, K.; Tao, J.; Fan, R.; Xu, Y.; Han, H.; Li, L.; Li, M.D. Association of STAT4 Polymorphisms with Hepatitis B Virus Infection and Clearance in Chinese Han Population. *Amino Acids* **2016**, *48*, 2589–2598, doi:10.1007/S00726-016-2283-3.
27. Zhao, X.; Jiang, K.; Liang, B.; Huang, • Xiaoqiang STAT4 Gene Polymorphism and Risk of Chronic Hepatitis B-Induced Hepatocellular Carcinoma., doi:10.1007/s12013-014-0205-0.
28. Liao, Y.; Cai, B.; Li, Y.; Chen, J.; Tao, C.; Huang, H.; Wang, L. Association of HLA-DP/DQ and STAT4 Polymorphisms with HBV Infection Outcomes and a Mini Meta-Analysis. *PLoS One* **2014**, *9*, doi:10.1371/JOURNAL.PONE.0111677.
